# Tips and rules for easy design of active microRNA-encoded peptides and complementary peptides

**DOI:** 10.1093/plphys/kiae493

**Published:** 2024-09-17

**Authors:** Mélanie Ormancey, Sabine Tourneur, Cécile Pouzet, Patrice Thuleau, Serge Plaza, Jean-Philippe Combier

**Affiliations:** Laboratoire de Recherche en Sciences Végétales, CNRS/UT3/INPT, 31320 Auzeville-Tolosane, France; Laboratoire de Recherche en Sciences Végétales, CNRS/UT3/INPT, 31320 Auzeville-Tolosane, France; Fédération de Recherche Agrobiosciences, Interactions et Biodiversité, UT3/CNRS, 31320 Auzeville-Tolosane, France; Laboratoire de Recherche en Sciences Végétales, CNRS/UT3/INPT, 31320 Auzeville-Tolosane, France; Laboratoire de Recherche en Sciences Végétales, CNRS/UT3/INPT, 31320 Auzeville-Tolosane, France; Laboratoire de Recherche en Sciences Végétales, CNRS/UT3/INPT, 31320 Auzeville-Tolosane, France; Micropep Technologies, 31320 Auzeville-Tolosane, France

Dear Editor,

Modern agriculture faces huge challenges, such as changing climatic conditions, which will have a major impact on productivity. In parallel, more and more chemicals are being banned from agronomy, due to environmental and health risks, increasing plant diseases and weed development. In this context, it becomes urgent to develop safe alternatives to chemicals to ensure sufficient productivity to feed the world's growing population. In recent years, peptide biology has attracted increasing interest. Recently, we characterized new peptide properties, with the identification of microRNA-encoded peptides (miPEPs; Lauressergues et al. 2015; 2022) and complementary peptides (cPEPs; Ormancey et al. 2023). miPEPs are natural peptides encoded by the primary transcripts of microRNAs (pri-miRNAs) increasing their transcription, through the direct interaction between miPEPs and their nascent mRNA (Lauressergues et al. 2015; 2022). Using this property of the interaction between a peptide and its nascent mRNA extrapolated to coding genes, cPEPs have been defined as peptides specifically enhancing the translation of their targeted protein, by facilitating the recruitment of ribosomes (Ormancey et al. 2023). These properties make these peptides potentially interesting for agronomy (Ormancey et al. 2021, 2023, 2024). However, identifying relevant miPEPs (which are encoded by the first ORF of pri-miRNAs) may not be so straightforward due to the lack of robust annotations of pri-miRNAs among plant species. Furthermore, the design and identification of cPEPs targeting homologous proteins in different species have never been described. We show here that the identification of active miPEPs is facilitated by the identification of miRNA-derived cPEPs. Indeed, miPEPs were identified as peptides encoded by the first ORF of pri-miRNAs. Consequently, the identification of miPEPs required the identification of the transcription start site of pri-miRNAs, using delicate approaches like RACE-PCR (Lauressergues et al. 2015), which is not necessary using miRNA-derived cPEPs. Moreover, we show here that it is possible to design cPEPs that are either highly specific for certain proteins or that target homologous proteins in different species.

Based on the ability of miPEPs to bind to their nascent mRNA, a property that was at the origin of the identification of cPEPs (Lauressergues et al. 2022; Ormancey et al. 2023), we designed a peptide corresponding to the second ORF (miPEP171b2) of *Medicago truncatula* pri-miR171b and another corresponding to a truncated version (truncated miPEP171b1) of miPEP171b (miPEP171b1 was used as positive control) (Supplementary Fig. S1). Interestingly, both peptides were active in increasing pri-miR171b expression (Fig. 1A). We then designed cPEPs of the pri-miRNA (cmiPEPs), i.e. complementary peptides derived from pri-miR171b sequences randomly chosen outside the ORFs (cmiPEP171b1-3; Supplementary Fig. S1), and showed that these peptides were also active (Fig. 1A). To validate these results, we performed similar approaches with different *Arabidopsis thaliana* pri-miRNAs, and peptides corresponding to either miPEPs from different ORFs, truncated miPEPs or cmiPEPs were all active in increasing the expression of their nascent pri-miRNA (Supplementary Fig. S2).

**Figure 1.**
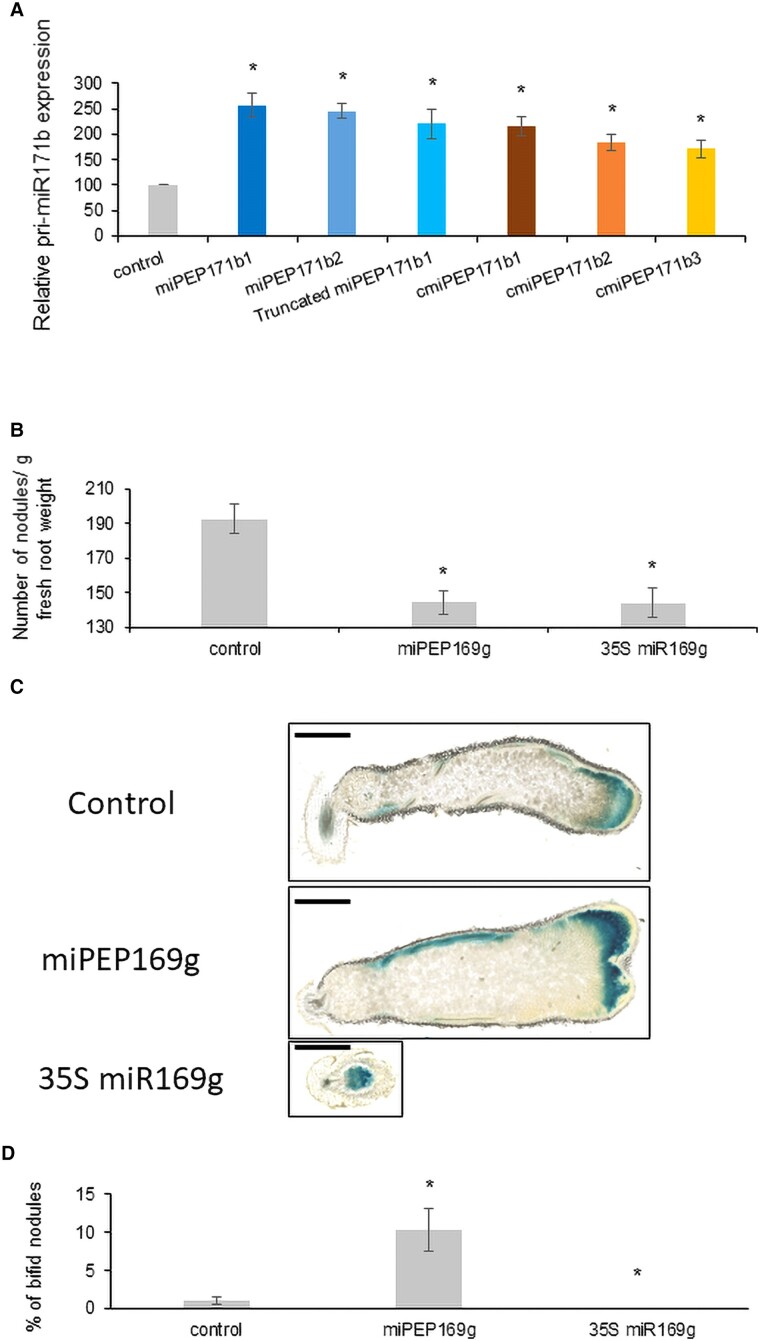
**A)** Relative expression of pri-miR171b in response to a treatment with 50 *µ*m of different peptides, in *Nicotiana benthamiana* leaves expressing pri-miR171b. **B** to **D)***M. truncatula* roots treated 3 times a week with 1 *µ*m of miPEP169g or transformed with 35S::miR169g construct, during 5 wk after inoculation with *Sinorhizobium meliloti*. **B)** Quantity of nodules per g of fresh root. **C)** 50-*µ*m sections of nodules. Bars represent 1 mm. **D)** Percentage of bifid nodules. Error bars represent SEMs, and asterisks indicate a significant difference between the test condition and the control according to Wilcoxon **A)** or Student **B**, **D)** test (**A**: *n* = 8; **B** to **D**: *n* = 40; *P* < 0.05).

Although miPEPs increase the expression of their own pri-miRNAs, the molecular processes involved cannot be compared to miRNA overexpression mechanisms, such as those using 35S promoter for example. Indeed, classical overexpression of a miRNA reduces target gene expression throughout the plant, therefore mimicking a multiple mutant of the target genes, whereas miPEPs decrease the expression of miRNA target genes only where and when the miRNA is expressed. To illustrate this, we generated *M. truncatula* plants overexpressing miR169g, known to be involved in nodulation (Combier et al. 2006), and compared them to miPEP169g-treated plants. Whereas both miRNA overexpression and miPEP treatment led to the formation of fewer nodules than in the control plants (Fig. 1B), the resulting nodules appeared completely different. Indeed, while miRNA overexpression led to small, nonmature nodules, miPEP169g treatment induced the formation of normal-sized nodules, but with a higher rate of bifid nodules (Fig. 1, C and D), which may be consistent with the role of the main target gene of miR169, MtNF-YA1, involved in meristem function during nodulation (Combier et al. 2006).

MiRNAs are sometimes difficult to detect. This is the case for example of miR169 in petunia, involved in petal formation (Cartolano et al. 2007). We therefore wondered whether a miPEP could be active, even if its nascent pri-miRNA is not detected. Thus, when petunias were treated with miPEP169, an acceleration of flowering was observed (Supplementary Fig. S3), indicating that the detection and/or the differential expression of corresponding miRNAs is not a sufficient argument for identifying relevant miPEPs.

Conversely, miPEPs may have unexpected effects. For example, when soybeans were treated with miPEP167c, whose corresponding miRNA is involved in nodulation (Wang et al. 2015), an increased leaf chlorophyll content was observed while there was no effect on nodule number (Supplementary Fig. S3).

Characterization of the miPEP-RNA interactions has led to the identification of cPEPs as peptides that enhance the translation of targeted coding genes, by interacting with the corresponding mRNA and ribosomes (Ormancey et al. 2023). To date, cPEPs have only been designed to target a single protein. To determine whether it is possible to design cPEPs targeting homologous proteins in different species, we first investigated the effect of mismatches between peptides and the targeted protein. To this end, we studied the impact of mismatches in a 10-amino acid cPEP targeting luciferase by successively replacing each amino acid in the cPEP with an alanine (Ormancey et al. 2023). Interestingly, changing C-terminal amino acids retained cPEP activity, whereas N-terminal amino acid modification abolished this activity (Fig. 2A). In a second step, we selected the EIN2 protein for its role in root development in order to design different cPEPs, one specific for *A. thaliana* EIN2 (cPEPein2), one specific for *M. truncatula* EIN2 (SKL protein; cPEPskl), and one targeting both proteins (cPEPein2/skl; Fig. 2B). Measurements of root development showed that the specific cPEP was active only on the targeted species, whereas the cPEP targeting the 2 homologous proteins was active on both plants (Fig. 2, C and D). This indicates that it is possible to design cPEPs that specifically target a protein from one species or cPEPs targeting homologous proteins in different species.

**Figure 2.**
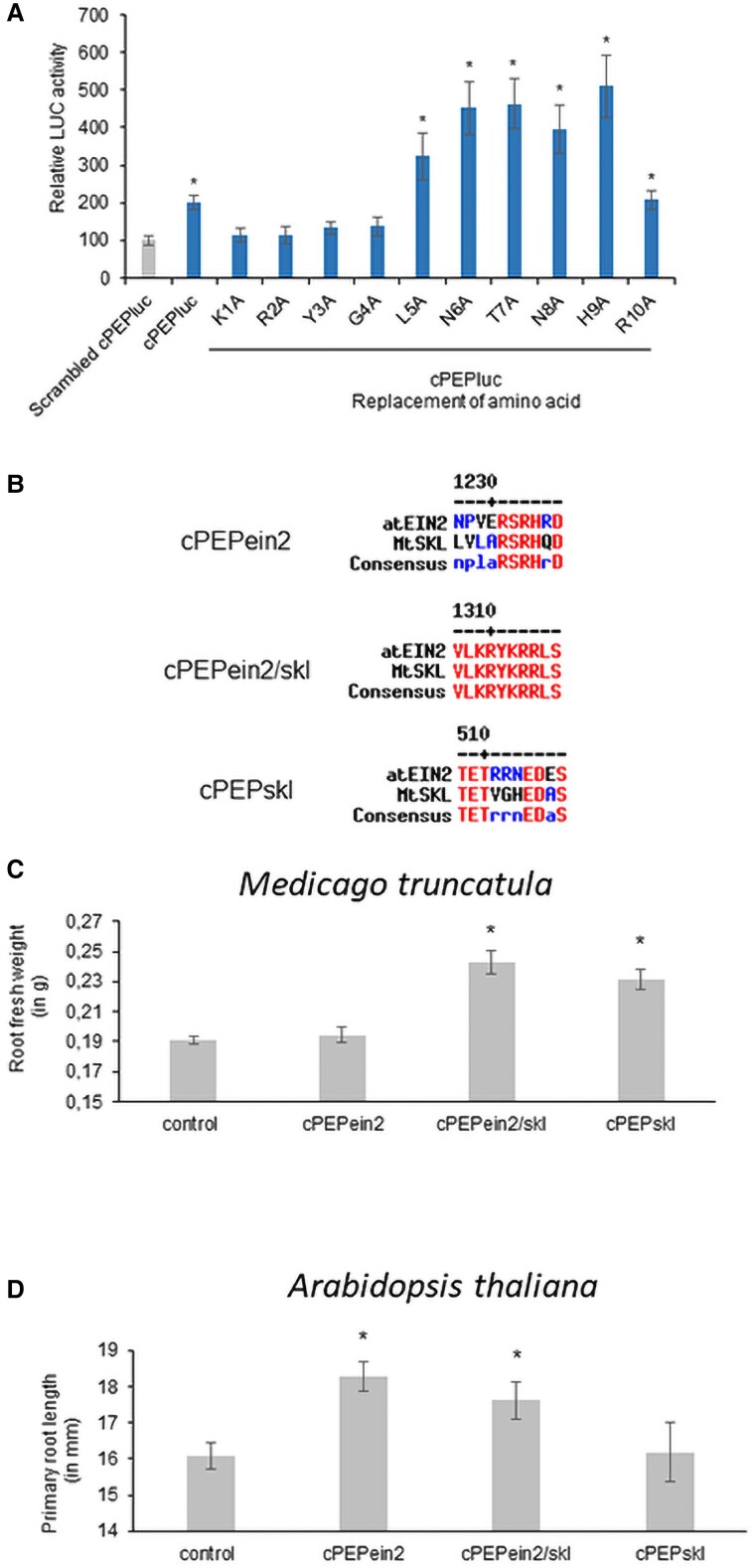
**A)** Luciferase activity of *A. thaliana* carrying LUC gene and treated for 24 h with 50 *µ*m of corresponding peptide. **B)** Sequence alignments using Multalin (Corpet 1988) of AtEIN2, MtSKL, and corresponding cPEPs. **C)** Fresh weight of *M. truncatula* roots treated 3 times a week during 2 wk with 50 *µ*m of the corresponding peptide. **D)** Length of *A. thaliana* primary roots treated 3 times a week during 2 wk with 50 *µ*m of the corresponding peptide. Error bars represent SEMs, and asterisks indicate a significant difference between the test condition and the control according to Wilcoxon **A)** or Student **C**, **D)** test (**A**: *n* = 8; **B** to **D**: *n* = 50; *P* < 0.05).

Finally, it is not always easy to predict the effect of a protein-targeting cPEP on a given phenotype based solely on the known phenotype of the corresponding mutant. Indeed, a cPEP targeting SHOT1 decreased heat stress tolerance (Supplementary Fig. S3), consistently with the phenotype of the shot1 mutant, which is resistant to heat stress (Kim et al. 2012). Conversely, when *A. thaliana* plants were treated with a cPEP targeting HSC70, the plants appeared more resistant to heat stress (Supplementary Fig. S3), which is the opposite of what we would have expected based on the heat tolerance of the *hsc70* mutant (Tiwari et al. 2020).

In conclusion, we show here that miPEPs and cPEPs are relevant tools to easily control miRNA and protein expression. In addition, we present new data to facilitate the design of active peptides, cPEPs or miPEPs, which could help decipher their mode of action without the need of transgenic plants and ultimately enable their use to replace chemicals in the context of sustainable agriculture.

## Acknowledgments

We thank Marc Knight (Durham University, UK) for Arabidopsis ABRE Luciferase lines.

## Supplementary data

The following materials are available in the online version of this article.

**Supplementary Figure S1.** Sequence of *M. truncatula* pri-miR171b.

**Supplementary Figure S2.** Expression of different *A. thaliana* pri-miRNAs in response to different peptides.

**Supplementary Figure S3.** Phenotypes of different plants treated with different peptides.

## Supplementary Material

kiae493_Supplementary_Data
