# Supplementary material for: Tips and rules for easy design of active microRNA-encoded peptides and complementary peptides
Source: Plant Physiol. 2024 Sep 17;196(4):2283–5. doi: 10.1093/plphys/kiae493 (PMC11637990; doi:10.1093/plphys/kiae493)
Supplement: kiae493_Supplementary_Data [file kiae493_supplementary_data.pdf]

```
attggtcaaacatacatacagtagcactagctggtttcattattccactatgcttcttcataggctctccaaattttgcaaaa
 I  G  Q  T  Y  I  Q  -  H  -  L  V  S  L  F  H  Y  A  S  S  -  A  L  Q  I  L  Q  N
  L  V  K  H  T  Y  S  S  T  S  W  F  H  Y  S  T  M  L  L  H  R  L  S  K  F  C  K
   W  S  N  I  H  T  V  A  L  A  G  F  I  I  P  L  C  F  F  I  G  S  P  N  F  A  K

ttgaaagagacatagtatatatatcttagcaaggagaaattcaggatattgaggatgaagattgaagagtaatcagtgatgaa
  -  K  R  H  S  I  Y  I  L  A  R  R  N  S  G  Y  -  G  -  R  L  K  S  N  Q  -  -
I  E  R  D  I  V  Y  I  S  -  Q  G  E  I  Q  D  I  E  D  E  D  -  R  V  I  S  D  E
 L  K  E  T  -  Y  I  Y  L  S  K  E  K  F  R  I  L  R  M  K  I  E  E  -  S  V  M  K

gaaagcaagcaaggtattggcgcgcctcaatttgaatacatggctataaaaatgcatcatatcagccatgtagtttgattgag
R  K  Q  A  R  Y  W  R  A  S  I  -  I  H  G  Y  K  N  A  S  Y  Q  P  C  S  L  I  E
 E  S  K  Q  G  I  G  A  P  Q  F  E  Y  M  A  I  K  M  H  H  I  S  H  V  V  -  L  S
  K  A  S  K  V  L  A  R  L  N  L  N  T  W  L  -  K  C  I  I  S  A  M  -  F  D  -  Q

ccgcgtcaatatcttgtttccatctccaa
 P  R  Q  Y  L  V  S  I  S
  R  V  N  I  L  F  P  S  P
   A  A  S  I  S  C  F  H  L
```

cmiPEP171b1

cmiPEP171b2

miPEP171b1

Truncated miPEP171b1

cmiPEP171b3

miPEP171b2

miR171b

Suppl. Figure S1: Sequence of *M. truncatula* pri-miR171b, translated in the three phases. Peptides used for treatments are coloured.

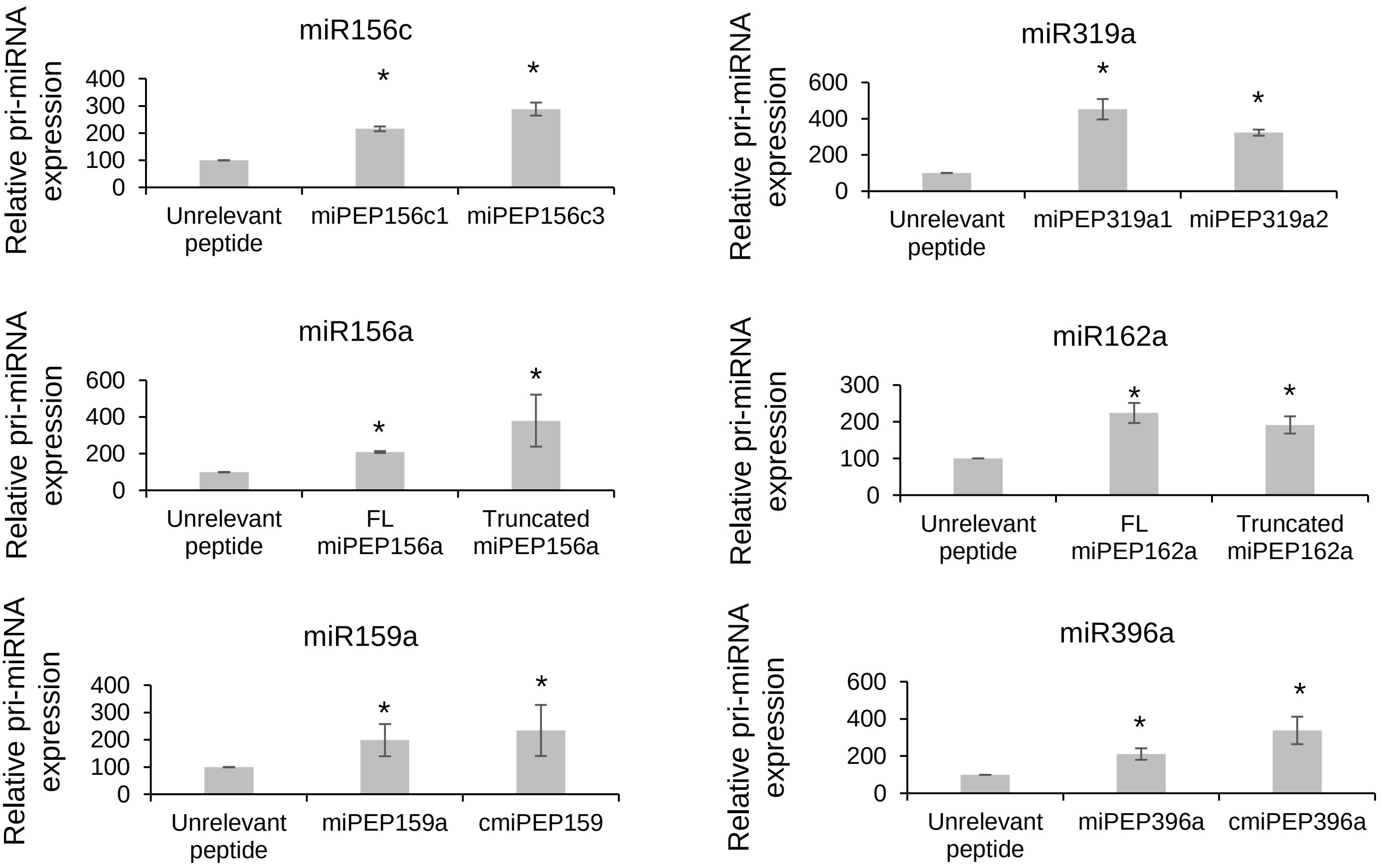

Suppl. Figure S2: Relative expression of different pri-miRNAs in response to 10 µM of different miPEPs, 24 h after treatment. Error bars represent SEMs, asterisks indicate a significant difference between the test condition and the control according to Wilcoxon test ($n = 8$; $p < 0.05$).

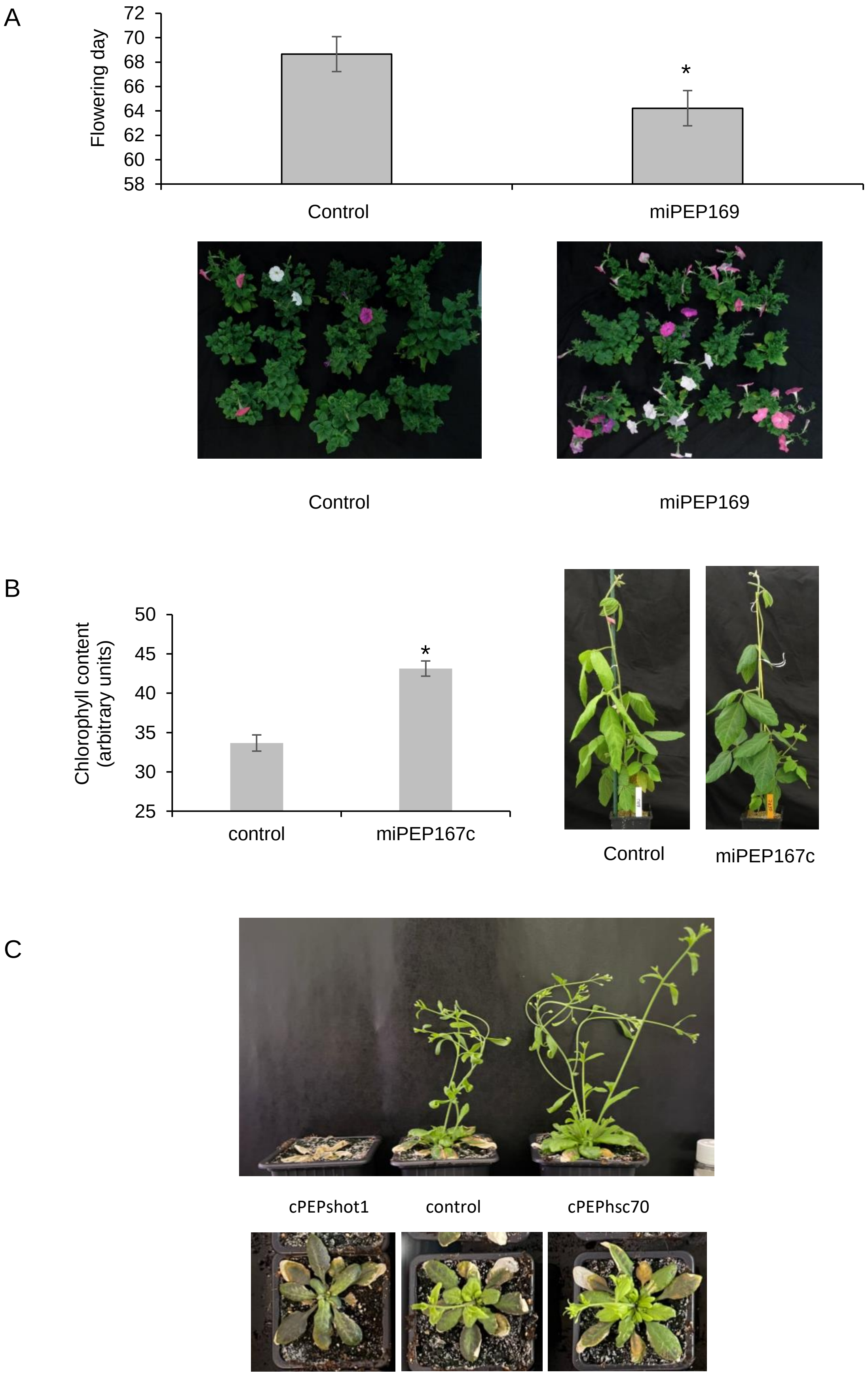

Suppl. Figure 3: A. Flowering day of petunia treated three times a week during two weeks with 50 µM of miPEP169. B. Chlorophyll content of Soybean plants watered three times a week during one month with 1µM of miPEP167c. C. Heat resistance of *A. thaliana* plants treated three times with 50 µM of corresponding peptide before a heat shock of 45 min at 45°C. Error bars represent SEMs, asterisks indicate a significant difference between the test condition and the control according to Student t-test (A: n = 50, B: n = 30; $p < 0.05$).

Methods

Biological material and growth conditions

Medicago truncatula Gaertn cv. Jemalong genotype A17 plants were cultivated on Fahraeus medium and inoculated with the *S. meliloti* strain RCR2001 pXLGD4 (GMI6526) (as described in Ormancey et al., 2023). Soybeans were cultivated as described in Couzigou et al., 2016. *Arabidopsis thaliana* Col-0 plants were grown on Jiffy up to 4 weeks-old in a growth chamber (22/20°C, 16 h/8 h Light/Dark, RH 80 %, ~ 75 µmol. m^{-2}. s^{-1}). ABRE-LUC seeds were provided by MR Knight (Durham University, UK), and were sterilized and sown on 96 wells plates containing 100 µL of MS/2 medium. For i*n vitro* experiments, surface-sterilized Col-0 seeds were sown onto ½ MS solid medium and stratified for 24h at 4°C in the dark (same conditions than above). Seedlings were vertically grown in a controlled growth chamber. *Nicotiana benthamiana* and *Petunia hybrida* seeds were sown on pots and cultivated in growth chamber. Agroinfiltration was performed as described in Ormancey et al., (2023).

Peptides

Peptides were synthesized by Smart Biosciences (www.smart-bioscience.com) and dissolved at 2-10 mM in water, aliquoted and conserved at - 80 °C. cPEPs were designed from translated nucleotide sequence of the target gene.

Name	Sequence
miPEP171b1	MLLHRLSKFCKIERDIVYIS
miPEP171b2	MKIEE
Truncated miPEP171b1	KIERDIVYIS
cmiPEP171b1	LVKHTYSSTSWFHYS
cmiPEP171b2	LVSLFHYASS
cmiPEP171b3	GSPNFAKLKET
MtmiPEP169g	MLSSSFGESSYSFLVLLFWQY
FL miPEP156a	MFCSIQCVARHLFPLHVREIKKATRAIKKGKTL
Truncated miPEP156a	TRAIKKGKTL
miPEP156c1	MKDNFPLLLRL
miPEP156c3	MREFWDKF
miPEP159a	MTWPLLSLSFLLSKYV
cmiPEP159a	NPRSHQNFL
FL miPEP162a	MVSGQEDSWLKLSSLCFLFLSLLDSLI
Truncated miPEP162a	SGQEDSWLKL
miPEP319a1	MNIHTYHHLLFPSLVFHQSSDVPNALSLHIHTYEYIIVVIDPFRITLAFR
miPEP319a2	MFQTLYLFIYIHTNILLLS
miPEP396a	MTLSVFFHSFLELQNFFRFFFFSFDISYA
cmiPEP396a	RVSSALHDP
cPEPein2	NPVERSRHRD
cPEPein2/skl	VLKRYKRRLS
cPEPskl	TETVGHEDAS
cPEPluc	KRYGLNTNHR
K1A	ARYGLNTNHR
R2A	KAYGLNTNHR
Y3A	KRAGLNTNHR
G4A	KRYALNTNHR
L5A	KRYGANTNHR
N6A	KRYGLATNHR
T7A	KRYGLNANHR
N8A	KRYGLNTAHR
H9A	KRYGLNTNAR
R10A	KRYGLNTNHA
Control	RNADAGRGIP
GmmiPEP167c	MKGVHHFFHHKYVGLRG
PhmiPEP169	MLHFFLGKLHFPKISMI
cPEPshot1	EHFGLHKKEL
cPEPhsc70	EFKRKHKKDI

Expression analyses

Gene expression quantification was performed by qRT-PCR. Levels of expression for the controls were set at 100. List of primers used. Primers for miR171b expression come from Lauressergues et al. (2015).

AtActin q5	GGTAACATTGTGCTCAGTGG
AtActin q3	CTCGGCCTTGGAGATCCACA
AtmiR156aq5	CTTCTCTTGCGTGCTCACTG
AtmiR156aq3	ACGAAGACAGGCCAAAGAGA
AtmiR156cq5	GATGAGGGAGTTTTGGGACA
AtmiR156cq3	CAGTGAGCACGCAAGAGAAG
AtmiR159aq5	ACTGCCTCGGGTAGATGAGA
AtmiR159aq3	CAGTGCATATGGCAGCAAAG
AtmiR162aq5	TGTGTTTCGTTTGATCCGATT
AtmiR162aq3	CCTCCAGCGACTCTCACTCT
AtmiR163q5	AGTTCCCGGTTCCTGAGAGT
AtmiR163q3	TAAATCCCCAAATGGGTTCA
AtmiR167aq5	TGTTGTGTTTCATGACGATGG
AtmiR167aq3	ACAGTCAACGGGTGAAACTG
AtmiR319aq5	TCATCGAGAGAGAGCTTCCTTGAGTC
AtmiR319aq3	TCCGGATCCAGAGGGAGCTCCCTTCAGT
AtmiR396aq5	CCTCACTCCCTCTTTCCACA
AtmiR396aq3	AGGGTCATGTAGAGCAGACGA

Peptide treatments

N. benthamiana plants were treated by spraying leaves 24 h before harvesting. For Luc assays, 100 µL of MS/2 liquid medium containing peptide was added to each well. 5 µL of luciferin was added and luciferase activity was read 30 min later using a spectrophotometer. *Medicago* plants were watered with 1 µM of peptide three times a week, beginning one week after germination. Inoculation was performed two weeks after germination and the experiment was stopped after 35 days. Concerning Arabidopsis root development, Col-0 seeds were grown *in vitro* as described above. Three days after sowing, seedlings were treated with 50 µM of the corresponding peptide every 2 days for 2 weeks. Seedlings were harvested 24h after the last treatment and scanned to quantify the primary root length using NeuronJ plugin of ImageJ. Floral buds of petunia were treated three times a week during two weeks with 50 µM of miPEP169. Soybean plants watered three times a week during one month with 1µM of miPEP167c, before measurement of chlorophyll with Chlorophyll Meter SPAD-502Plus. *Arabidopsis thaliana* plants were treated three times with 50 µM of corresponding peptide before a heat shock of 45 min at 45°C. Pictures were taken 15 days after heat shock.

Statistical analyses

The mean values of relative gene expression or phenotypical parameters were compared by using the Wilcoxon or the Student t-test. The error bars represent the standard error of the mean (SEM). The asterisks indicate significant differences ($p<0.05$). In all the figures, "n" corresponds to biological replicates.

References

Couzigou JM, André O, Guillotin B, Alexandre M, Combier JP. Use of microRNA-encoded peptide miPEP172c to stimulate nodulation in soybean. New Phytologist. 2016. 211:379-381.

Lauressergues D, Couzigou JM, San Clemente H, Martinez Y, Dunand C, Bécard G, Combier JP. Primary transcripts of microRNAs encode regulatory peptides. Nature. 2015. 520:90-3.

Ormancey M, Guillotin B, Merret R, Camborde L, Duboé C, Fabre B, Pouzet C, Impens F, Van Haver D, Carpentier MC, San Clemente H, Aguilar M, Lauressergues D, Scharff L, Pichereaux C, Burlet-Schiltz O, Bousquet-Antonelli C, Gevaert K, Thuleau P, Plaza S, Combier JP. Complementary peptides represent a credible alternative to agrochemicals by activating translation of targeted proteins. Nature Communications. 2023. 14:254.
